# Frenkel line crossover of confined supercritical fluids

**DOI:** 10.1038/s41598-019-49574-3

**Published:** 2019-10-16

**Authors:** Kanka Ghosh, C. V. Krishnamurthy

**Affiliations:** Department of Physics, Indian Institute of Technology Madras, Chennai, 600036 India

**Keywords:** Chemistry, Physics

## Abstract

We investigate the temperature evolution of dynamics and structure of partially confined Lennard Jones (LJ) fluids in supercritical phase along an isobaric line in the P-T phase diagram using molecular dynamics simulations. We compare the Frenkel line (FL) crossover features of partially confined LJ fluids to that of the bulk LJ fluids in supercritical phase. Five different spacings have been chosen in this study and the FL crossover characteristics have been monitored for each of these spacings for temperatures ranging from 240 K to 1500 K keeping the pressure fixed at 5000 bar. We characterize the FL crossover using density of states (DoS) function and find that partially confined supercritical fluids (SCF) exhibit a progressive shift of FL crossover point to higher temperatures for smaller spacings. While the DoS perpendicular to the walls shows persistent oscillatory modes, the parallel component exhibits a smooth crossover from an oscillatory to non-oscillatory characteristics representative of FL crossover. We find that the vanishing of peaks in DoS parallel to the walls indicates that the SCF no longer supports shear mode excitations and could serve as an identifier of the FL crossover for confined systems just as is done for the bulk. Layer heights of density profiles, self-diffusivity and the peak heights of radial distribution function parallel to the walls also feature the FL crossover consistent with the DoS criteria. Surprisingly, self-diffusivity undergoes an Arrhenius to super-Arrhenius crossover at low temperatures for smaller spacings as a result of enhanced structural order evidenced via pair-excess entropy. This feature, typical of glass-forming liquids and binary supercooled liquids, is found to develop from the glass-like characteristic slowdown and strong caging in confined supercritical fluid, evidenced via mean squared displacement and velocity autocorrelation function respectively, over intermediate timescales.

## Introduction

Over the past few years, experiments and molecular dynamics simulations have confirmed the existence of two distinct phases in the bulk supercritical fluids (SCF), before and after crossing Frenkel line (FL)^1–5^. The microscopic dynamics and structural features of the fluids evolved into either “liquidlike” or “gaslike” phases as the FL was traversed. This FL crossover is associated with a smooth transition in the particle dynamics and structural properties of the bulk supercritical fluids. In a recent experimental study of Frenkel line crossover in bulk supercritical Neon, C. Prescher and co-authors^6^ found the width of this crossover to be smaller than 10% in both pressure and temperature. Our studies on partially confined supercritical LJ fluids^7,8^ opened up the possibility of the existence of an amorphous phase in addition to the liquidlike and gaslike phases in supercritical LJ fluids. As partial confinement has been found to strongly influence the dynamics and induce significant changes in the structure, its role on the characteristics associated with the Frenkel line and its vicinity are worth investigating.

In the context of liquidlike and gaslike regimes of supercritical fluids, it is important to mention the occurrence of Widom line, validated via both inelastic x-ray scattering and molecular dynamics simulations^9^. We must note here the fundamental difference between the Widom and the Frenkel line. Widom line in the phase diagram is defined by the locus of the maxima^10^ of the thermodynamic response functions, whereas Frenkel line is associated with a crossover of fundamental microscopic structural and dynamical characteristics in supercritical fluids. While Frenkel line has been found to be present up till arbitrary high pressure and temperature, Widom line seems to exist only near the critical point^1^ and had been found not to obey the corresponding states principle^10^.

Frenkel line is characterized by a demarcation line of finite width^6^ (<10% in P and T) that defines a crossover from damped oscillatory dynamics and liquidlike structural ordering to non-oscillatory dynamics and gaslike diffusion dominated behavior of SCFs. We seek answers to two related questions:

- How does the dynamics evolve over various time scales and influence the structure in the vicinity of the FL?
- How would partial confinement modify the crossover characteristics of FL?

To answer these questions, we investigate the dynamics and structure of confined SCFs along the isobaric line (P = 5000 bar) encompassing a wide range of temperature points from 240 K to 1500 K through molecular dynamics simulations. We explore 5 different spacings for this study: H = 70 Å (≈21*σ*), 30 Å (≈9*σ*), 20 Å (≈6*σ*), 10 Å (≈3*σ*) and 6.8 Å (=2*σ*). For each spacing, temperatures ranging from 240 K to 1500 K have been simulated keeping pressure fixed at 5000 bar. The densities of the confined systems are maintained to be identical to that in the bulk phase corresponding to the chosen P and T. To keep the density fixed, the confined domains are made rectangular parallelepiped with varying lateral dimensions. A set of 10^5^ particles interacting with truncated and shifted Lennard Jones (LJ) potential mimicking argon ($\varepsilon$/k_*B*_ = 120 K, *σ* = 3.4 Å, cutoff = 20 Å ~ 6*σ*) are simulated. NPT ensembles are employed to simulate SCF in bulk phase while NVT ensembles are used to simulate SCFs under atomistic boundaries. Atomistic walls are modelled perpendicular to the z-axis and periodic boundary conditions are employed along other two unconfined directions (x, y). The wall atoms are attached to fcc lattice sites by harmonic springs. The spring constant (k) for these springs are fixed to a value of 1000 eV/Å^2^ which mimic rigid walls^7,8^, by restricting the mean squared displacement (MSD) of the wall atoms with respect to their lattice sites to a very low value. The root mean squared displacement (RMSD) of wall particles are restricted to be 40 times and 100 times smaller than a typical distance traversed by a fluid particle between two collisions^1^ (~1 Å), at 1500 K and 300 K respectively.

Velocity-Verlet algorithm with a time step (Δt) of 0.0001 ps is used to equilibrate the system up to 50 ps followed by a 1000 ps production run to calculate and analyze the dynamics and structural properties of interests. All molecular dynamics simulations described in this article are performed using Large scale atomic/molecular massively parallel simulator (LAMMPS)^11^.

## Results and Discussion

The distribution of vibrational modes of the system is obtained from the density of state (DoS) function, S(*ν*)^12^. The number of modes at some frequency *ν* can be calculated as^12^

1 $$S(\nu )=\frac{2}{{k}_{B}T}\,\mathop{\sum }\limits_{k=1}^{N}\,\mathop{\sum }\limits_{l=1}^{3}\,{m}_{j}{s}_{j}^{l}(\nu )$$

where, ${s}_{j}^{l}(\nu )$ = spectral density of atom j in l^*th*^ coordinate and is extracted from the square of the Fourier transform of velocities as

2 $${s}_{j}^{l}(\nu )=\mathop{\mathrm{lim}}\limits_{\tau \to \infty }\,\frac{1}{2\tau }\,{|{\int }_{-\tau }^{\tau }{v}_{j}^{l}(t){e}^{-i2\pi \nu t}dt|}^{2}$$

Implementing Wiener-Khintchine theorem, the atomic spectral density reduces to the Fourier transform of autocorrelation function^12^.

3 $$\begin{array}{rcl}{s}_{j}^{l}(\nu ) & = & \mathop{\mathrm{lim}}\limits_{\tau \to \infty }\,\frac{1}{2\tau }{|{\int }_{-\tau }^{\tau }{v}_{j}^{l}(t){e}^{-i2\pi \nu t}dt|}^{2}\end{array}$$

4 $$\begin{array}{rrl} & = & \mathop{\mathrm{lim}}\limits_{\tau \to \infty }\,\frac{1}{2\tau }\,{\int }_{-\tau }^{\tau }\,{\int }_{-\tau }^{\tau }\,{v}_{j}^{l}(t){v}_{j}^{l}(t+t^{\prime} )dt^{\prime} {e}^{-i2\pi \nu t}dt\end{array}$$

5 $$\begin{array}{rcl} & = & \mathop{\mathrm{lim}}\limits_{\tau \to \infty }\,{\int }_{-\tau }^{\tau }\,{z}_{j}^{l}(t){e}^{-i2\pi \nu t}dt\end{array}$$

where, ${z\,}_{j}^{l}$(t) = Velocity autocorrelation function (VACF).

Thus, in practice, DoS can be readily computed from the temporal Fourier transform [Z($\omega$)] of normalized VACF [Z(t)] and is defined as

6 $$Z(\omega )=\frac{1}{2\pi }\,{\int }^{}\,Z(t)\exp (\,-\,i\omega t)dt$$

where $\omega =2\pi \nu$ is the angular frequency. Z($\omega$) has been found to be a sensitive quantity^13,14^ for probing the dynamics of fluids in frequency domain. For a typical gas phase, Z($\omega =0$) $>$ 0 and decays monotonically. The finite intercept of DoS at $\omega =0$ corresponds to the diffusive modes^12,13^. For liquids, along with diffusive modes on long timescales Z($\omega =0$) $>$ 0, there exists nondiffusive modes arising out of caging effects on shorter timescales giving rise to a structure in Z($\omega$) for $\omega  > 0$: a maximum followed by a decay at higher frequencies.

We evaluate DoS for the bulk SCF as a function of temperature along the isobaric line defined by P = 5000 bar, to determine the behavior of the DoS across the FL. Following the idea of Shiang-Tai Lin *et al*.^12^, we use the DoS to monitor the gaslike to liquidlike features exhibited by SCF across the FL. The 2PT model of DoS, originally proposed by Shiang-Tai Lin *et al*.^12^ via decomposing the solid-like and gas-like contributions of DoS, has recently been used successfully to understand a variety of properties of fluids such as identifying rigid to non-rigid transition in dynamics^15^, studying the pressure evolution of the transverse collective modes of liquid Al^16^ and calculating entropy of water confined within graphene plates^17^ to name a few.

**Figure 1:**
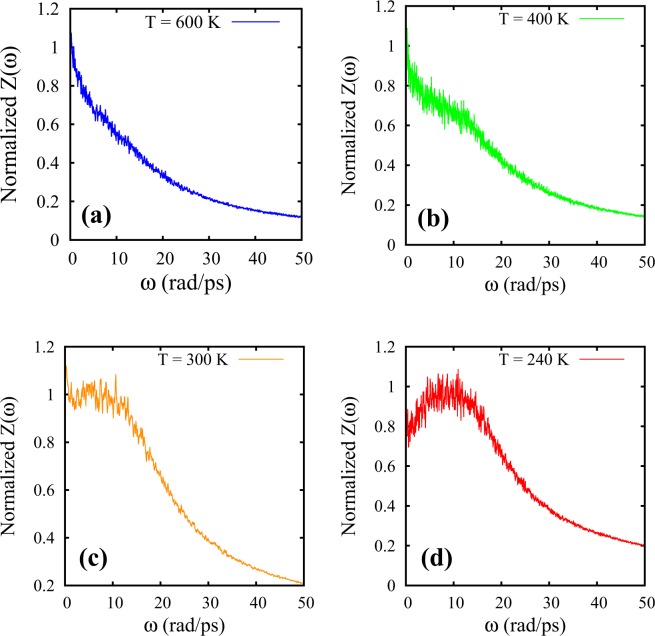
The DoS (Z($\omega$)) for bulk supercritical Argon at P = 5000 bar. (**a**–**d**) Represent DoS of bulk SCF at T = 600 K, 400 K, 300 K and 240 K respectively.

The existence of non-diffusive vibrational modes can be inferred by the manifestation of the peak in the DoS at non-zero frequency as temperature is lowered from the gaslike phase at 600 K (Fig. 1). In other words, bulk SCF gradually develops its non-diffusive vibrational modes as we decrease the temperature from 600 K. FL crossover in bulk SCF is observed around 600 K at P = 5000 bar as the DoS completely loses vibrational component around 600 K. This is consistent with the complementary approach of determining FL crossover using VACF, as deduced in our previous work^7^. We note that while the peak in the DoS for $\omega  > 0$ corresponds to damped-oscillatory VACF (Fig. 1(d)), the monotonic decay in Z ($\omega$) for $\omega \geqslant 0$ corresponds to a non-oscillatory VACF (Fig. 1(a)).

Following the seminal theoretical work of T. Gaskell *et al*.^18,19^, we note that the DoS spectra of bulk liquids carry the signature of both transverse and longitudinal mode excitations with the transverse mode having the lower frequency response. A. I. Chumakov *et al*.^20^, also correlated the low-frequency peak in the DoS to the emergence of a shear propagation in the glass through their experimental study. Further, in a recent theoretical work^21^ on the exponential mode analysis of time correlation functions, different relaxation processes were identified and eventually the low frequency DoS modes of liquid metals had been attributed to the transverse acoustic modes. Studies on supercritical fluids^5,22,23^ had associated FL crossover with a complete loss of the ability to support shear modes.

We recall that even dilute gases exhibit collective motion under confinement^24^. We found that the DoS for confined gas^24^ exhibited a peak for $\omega  > 0$. It is therefore quite possible that the DoS for SCF would be influenced by confinement. We examine the changes in the parallel (Z_*xy*_ ($\omega$)) and perpendicular (Z_*z*_ ($\omega$)) components of the DoS for confined SCF from 240 K to 1500 K at P = 5000 bar for 5 different spacings (H = 70 Å (≈21*σ*), 30 Å (≈9*σ*), 20 Å (≈6*σ*), 10 Å (≈3*σ*) and 6.8 Å (=2*σ*)). The spacing of 70 Å is chosen as a representative spacing close to the bulk as the particle distribution across the spacing exhibits identically flat distribution similar to the bulk for almost the whole spacing except the vicinity of the walls. The rest of the spacings are selected as strong structural inhomogeneities are found to occur at these spacings^7^.

**Figure 2:**
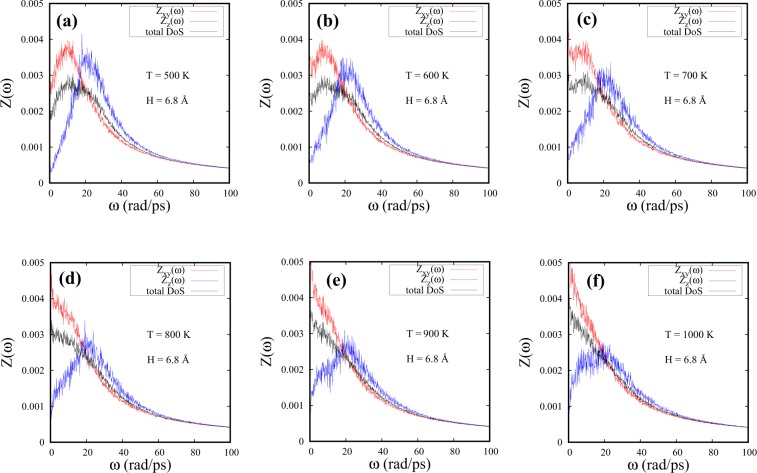
DoS parallel to the walls [Z_*xy*_($\omega$)], perpendicular to the walls [Z_*z*_($\omega$)] and the total DoS of SCF as a function of temperature for H = 6.8 Å (=2*σ*). The corresponding temperatures are (**a**) T = 500 K, (**b**) T = 600 K, (**c**) T = 700 K, (**d**) T = 800 K, (**e**) T = 900 K and (**f**) T = 1000 K.

The DoS plots associated with the parallel (xy) and perpendicular (z) velocity components for a confined spacing of H = 6.8 Å are shown as a function of temperature in Fig. 2. The total DoS is included for comparison.

It is interesting to note that the peak-like structure in Z_*z*_($\omega$) persists even at 1000 K with the peak location fairly independent of temperature. Strong confinement has been shown to lead to short-lived collective excitations^24^ and together with the high fluid density, leads to a weak temperature dependence. These excitations are longitudinal in nature. We observe that total DoS is dominated by the parallel component of DoS (Z_*xy*_($\omega$)) and the peaks in both total DoS and Z_*xy*_($\omega$) at a non-zero finite frequency, representative of non-diffusive vibrational modes, are seen to undergo a crossover to a monotonically decaying DoS as we increase the temperature. At T = 1000 K (Fig. 2(f)), Z_*xy*_($\omega$) and the total DoS show monotonically decaying feature with a complete loss of oscillatory modes parallel to the walls for H = 6.8 Å.

**Figure 3:**
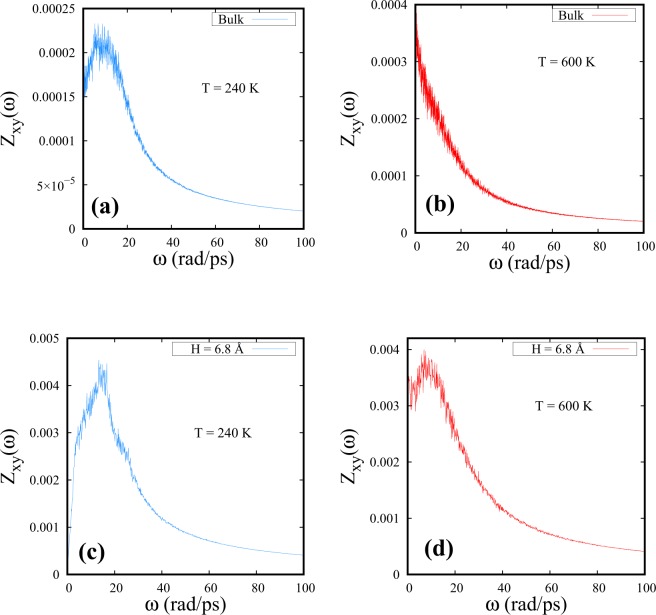
DoS parallel to the walls (Z_*xy*_($\omega$)) for bulk (**a**,**b**) and for H = 6.8 Å spacing (**c**,**d**) at T = 240 K (**a**,**c**) and T = 600 K (**b**,**d**).

Figure 3 shows that the peak in the Z_*xy*_($\omega$) component of DoS is disappearing at much higher temperatures under confinement than in the bulk. It can be seen from Fig. 3 that, at 240 K, there is a slight but distinct shift in the peak to higher frequencies for the confined case (Fig. 3(c)) when compared with that of the bulk (Fig. 3(a)). It seems likely that the shift of the peak to a higher frequency could be due to increasing “structural order”. There is also a shoulder appearing at higher frequencies which has been found coinciding with the frequency of the second peak in Z_*z*_($\omega$) (See Supplementary Fig. S3 for details). This shoulder in Z_*xy*_($\omega$) seems to exist for T = 240 K, 260 K and 280 K, where two-peak structures of Z_*z*_($\omega$) are also found to be present (Fig. S3 in Supplementary Information). At low temperatures and in a strongly confined SCF, wall-mediated collisions are found to influence the parallel component of DoS significantly. At 600 K, we observe that the bulk SCF loses all the oscillatory modes parallel to the walls with monotonically decaying Z_*xy*_($\omega$) at $\omega  > 0$ (Fig. 3(b)). The smallest spacing studied in this article, H = 6.8 Å, however, shows persistent oscillatory modes with a peak at a finite frequency in Z_*xy*_($\omega$) (Fig. 3(d)). Following Figs 2 and 3 we find that the FL crossover is found to occur at ≈1000 K for H = 6.8 Å.

Although the total DoS closely follows the trends shown by the parallel component of DoS, as shown in Fig. 2, the vanishing of the peak in the parallel component of Z_*xy*_($\omega$) appears to be a more appropriate indicator of the FL crossover. The vanishing of the peak in Z_*xy*_($\omega$) indicates that the fluid no longer supports shear mode excitations and could serve as an identifier of the FL crossover just as is done in the bulk^5,22,23^.

**Figure 4:**
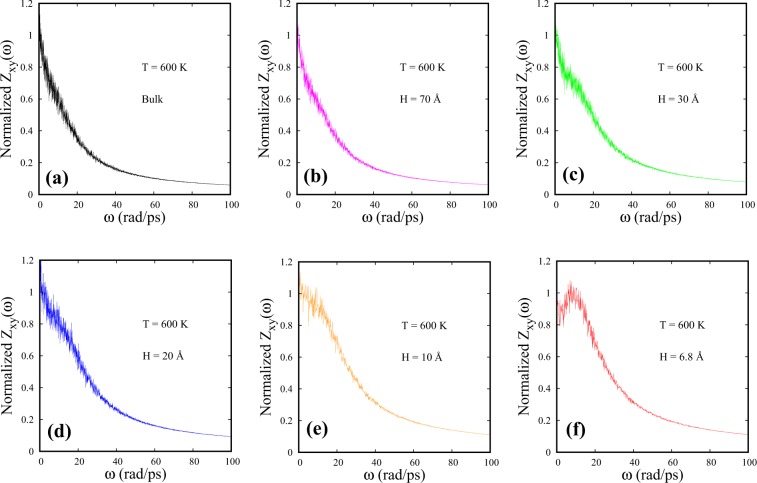
DoS parallel to the walls [Z_*xy*_($\omega$)] of SCF as a function of spacing at T = 600 K. The corresponding spacings are: (**b**) H = 70 Å, (**c**) H = 30 Å, (**d**) H = 20 Å, (**e**) H = 10 Å and (**f**) H = 6.8 Å. The bulk Z_*xy*_($\omega$) has been shown in (**a**).

From the trends in Figs 2 and 3 a significant result emerges: namely, partial confinement leads to the FL crossover temperatures towards values higher than that in the bulk. As the spacings (H) are reduced, the crossover gradually seems to shift to higher temperatures. In Fig. 4, DoS [Z_*xy*_($\omega$)] of SCF as a function of spacing is presented at a fixed temperature of 600 K. While bulk DoS [Z_*xy*_($\omega$)] shows a monotonically decaying feature at 600 K, partial confinement with H = 6.8 Å lead to prominent peak-like structures at non-zero frequencies signaling the presence of oscillatory modes, at the same temperature.

Partial confinement with H = 30 Å, 20 Å and 10 Å are found to exhibit relatively weaker oscillatory modes in DoS at 600 K (Fig. 4).

**Figure 5:**
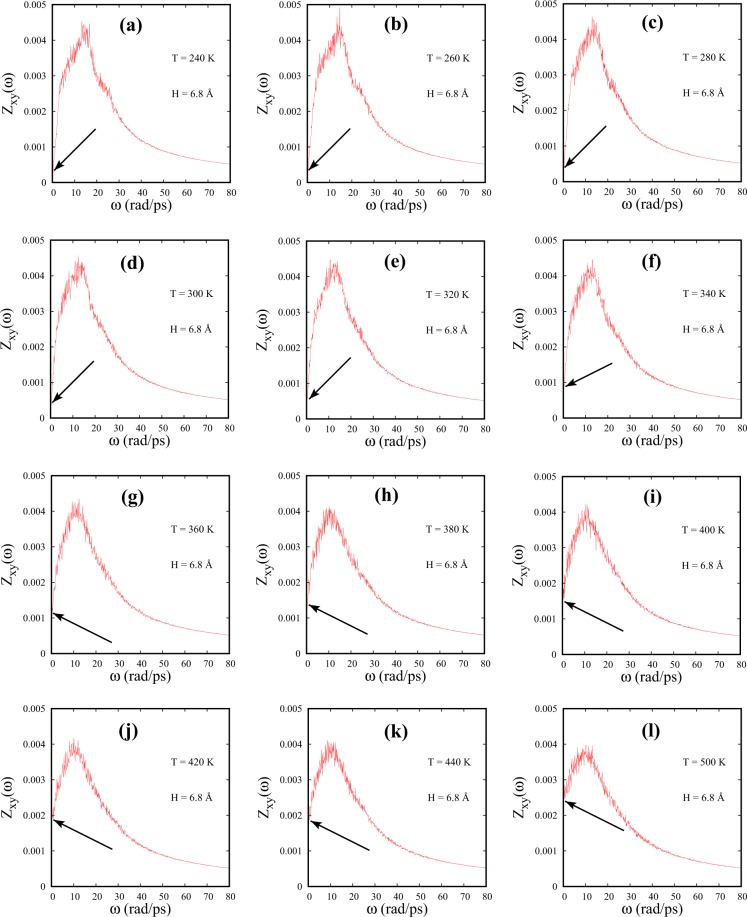
DoS parallel to the walls [Z_*xy*_($\omega$)] of SCF is presented for different temperatures for H = 6.8 Å system. The intercepts at $\omega =0$ are marked using arrows. The corresponding temperatures are (**a**) 240 K, (**b**) 260 K, (**c**) 280 K, (**d**) 300 K, (**e**) 320 K, (**f**) 340 K, (**g**) 360 K, (**h**) 380 K, (**i**) 400 K, (**j**) 420 K, (**k**) 440 K and (**l**) 500 K.

We note that the positive finite intercepts of DoS at $\omega =0$ indicate the presence of diffusive modes in fluids with diffusivity being proportional to Z($\omega$) at $\omega =0$^12^. Figure 4 shows diffusive modes in Z_*xy*_($\omega$) for both bulk and confined SCF at 600 K. To further investigate the diffusive modes parallel to the walls in strong confinement, we monitor the temperature variation of the intercepts of Z_*xy*_($\omega$) at $\omega =0$ for H = 6.8 Å (Fig. 5). At lower temperatures (240 K $\leqslant$ T $\leqslant$ 340 K), Z_*xy*_($\omega$) almost goes to zero at $\omega =0$, resembling a solid-like DoS^12^ with almost negligible diffusive properties (Fig. 5(a–f)). As temperature is increased beyond 340 K, the intercepts of Z_*xy*_($\omega$) at $\omega =0$ progressively shift to higher values signaling a gradual increase of diffusivity of fluid particles parallel to the walls with liquidlike DoS (Fig. 5(g–l)). Strong confinement, manifest via wall-mediated collisions, tends to influence the parallel component of DoS strongly at lower temperatures. This causes the SCF to display almost solid-like vibrational modes with negligible diffusivity parallel to the walls. This feature indicates an unusual diffusive behavior of confined SCF than what one would expect in the liquidlike regime of bulk SCF.

**Figure 6:**
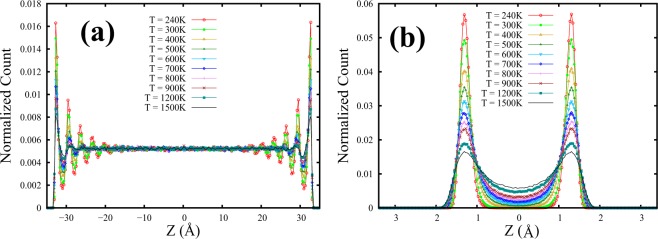
Particle distribution profiles of confined SCF perpendicular to the walls (along z) as a function of temperature. (**a**,**b**) Represent confined SCF systems with H = 70 Å (≈9*σ*) and 6.8 Å (=2*σ*) respectively.

We had seen earlier that partial confinement leads to intriguing evolution of particle dynamics and emerging structures at a fixed temperature far from any phase change^7,8^. We now look at the role of temperature on the dynamics and structure of partially confined SCF. To begin with, we look at the structural features perpendicular to the walls - in terms of the local particle density variations across the thickness, as a function of temperature. Figure 6 shows the evolution of layering across the spacing as a function of temperature for two extreme spacings covered in this study. Particle number density is found to vary strongly in the direction perpendicular to the confining walls leading to layering, giving rise to peaks in the number density distribution along the z-axis. The peak height of each layer together with the manner in which particles are packed within that layer contain valuable information about the evolution of the structural ordering across FL. As the peak near the wall is a manifestation of strong wall-particle correlation and seems to exist even in the gaslike phase, we track the peak height of the layer closest to the wall as a function of temperature. Supplementary Figure S1 (shown in the Supplementary Material) describes the temperature evolution of the peak heights (h) for the layer closest to the wall for two extreme spacings H = 6.8 Å and H = 70 Å. It is observed that the peak height of the layer closest to the wall decays faster for H = 6.8 Å compared to the H = 70 Å spacing (Fig. S1 in Supplementary Material).

**Figure 7:**
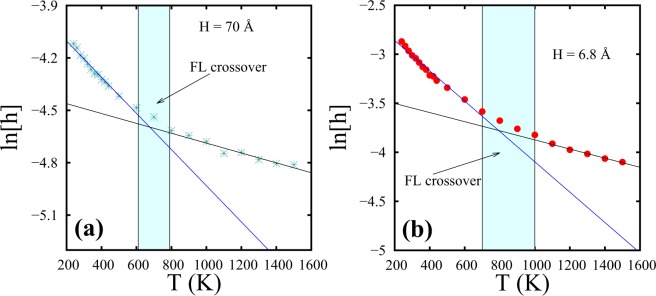
Peak heights (h) of the layer closest to the wall as a function of temperature for supercritical Argon for two different spacings. Liquidlike and gaslike regimes are characterized by distinct slopes and FL crossover regions are identified at around 600–800 K for (**a**) H = 70 Å and at around 700–1000 K for (**b**) H = 6.8 Å. Layer height (h) has been represented in logscale.

To further investigate this feature, we re-plot the peak height variation with temperature using logscale as shown in Fig. 7. Figure 7 describes the liquidlike and gaslike regimes of SCF, for two spacings H = 70 Å and H = 6.8 Å, characterized by distinct slopes indicating the FL crossover. The crossover temperature can be identified to lie within an approximate range set by the trends of the two data segments as shown by a colored band in Fig. 7(a,b). This structural crossover is observed at around 600–800 K for H = 70 Å (Fig. 7(a)) spacing and at 700–1000 K for H = 6.8 Å (Fig. 7(b)).

We recall that motion perpendicular to the walls becomes sub-diffusive. To study the dynamics across the FL, we therefore calculate the self-diffusion coefficients parallel to the walls. For comparison, self-diffusion coefficients of bulk SCF has also been computed. Supplementary Fig. S2 (shown in Supplementary Material) shows the variation of self-diffusion coefficients (D), parallel to the walls, as a function of inverse of temperature for different confined spacings.

**Figure 8:**
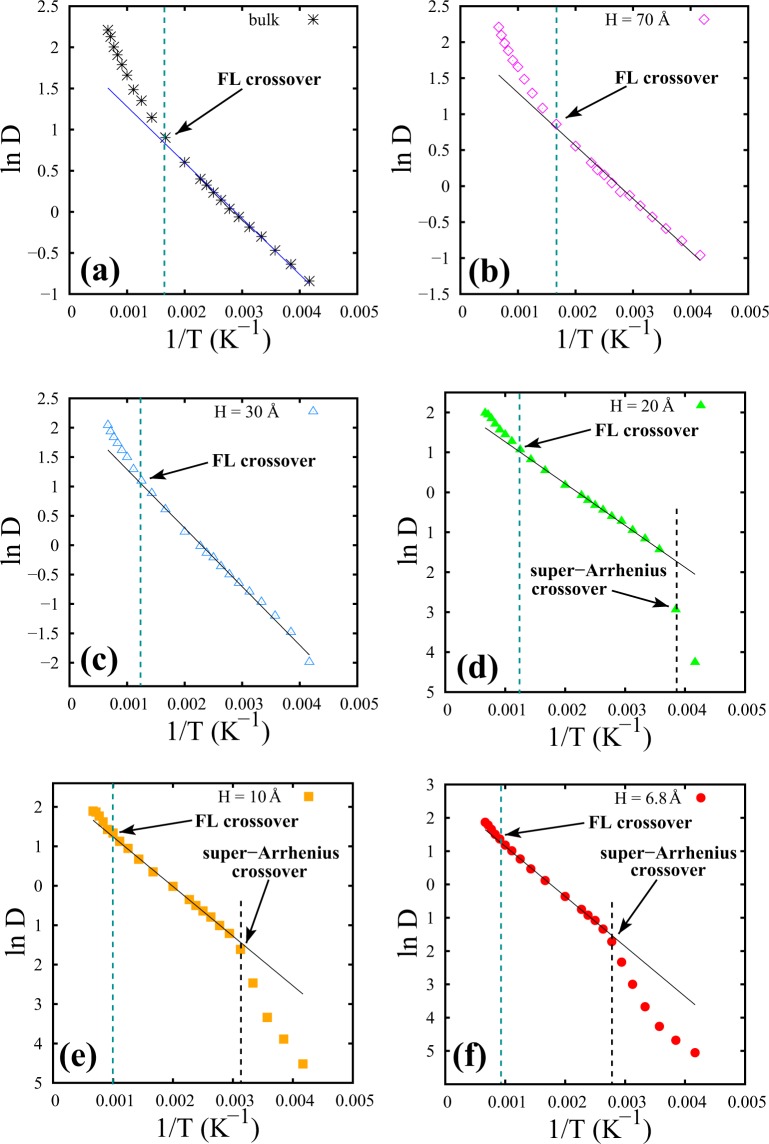
Self-diffusion coefficients (D) of SCF, parallel to the walls, as a function of temperature. (**a**–**f**) Represent systems correspond to bulk, H = 70 Å, 30 Å, 20 Å, 10 Å and 6.8 Å spacings respectively. FL crossover, Arrhenius behavior and transition to super-Arrhenius behavior are marked for these systems.

We observe from Supplementary Fig. S2 (shown in Supplementary Material) that the trends of self-diffusion coefficients (D) for different H diverge as the temperature is lowered. To further characterize the temperature dependence of D, we examine the Arrhenius behavior of D in the liquidlike regime of SCF as a function of temperature. Following V. V. Brazhkin *et al*.^1^, who found that D in the bulk phase followed Arrhenius law, exp(−E_*a*_/k_*B*_T), where E_*a*_ is the effective activation energy, we examine the behaviour of D in the liquidlike regime of the partially confined SCF. Figure 8 presents the variation of D as a function of temperature for bulk as well as for different spacings of the partially confined SCF.

As VACF and DoS show a different crossover in confined SCF than that of the bulk SCF, we expect a variation of D as a function of temperature at P = 5000 bar. Figure 8 shows that Arrhenius behaviour exists for a limited temperature regime. The liquidlike to gaslike FL crossover sets the limit at the high temperature end.

Surprisingly, for smaller spacings (6.8 Å ≤ H ≤ 20 Å), an unusual deviation from Arrhenius behavior seems to exist at low temperatures (Fig. 8). This deviation of the self-diffusion coefficient (D) from Arrhenius behavior as a function of temperature is termed as “super-Arrhenius” behavior. This is consistent with our earlier estimation of the diffusive modes from DoS parallel to the walls as shown in Fig. 5. We recall that at lower temperatures, SCF shows solid-like vibrational modes with an abrupt decrease in diffusivity parallel to the walls (Fig. 5(a–f)) for H = 6.8 Å. Such super-Arrhenius behaviour has been found in bulk supercooled liquids^25^ and binary Lennard Jones mixtures^26^. Also, NMR and quasielastic neutron scattering study revealed a similar Arrhenius (strong) to super-Arrhenius (fragile) crossover for supercooled confined water^27^. It is worth noting that the predictions made in the current work on confined fluids interacting with simple LJ potentials closely resemble experimental observations on the more complex fluid such as supercooled confined water^27^. It appears that the Arrhenius to super-Arrhenius behaviour could be a more generic feature arising out of the interplay of confinement and temperature effects rather than on the details of the interactions. Here, we emphasize that the Arrhenius to super-Arrhenius crossover takes place for smaller spacings within the supercritical phase of Argon at lower temperatures. The temperatures corresponding to this crossover from Arrhenius behavior sets the lower and upper limits. These two temperature limits and the activation energies associated with the Arrhenius behaviour are presented in Table 1 for various confinement spacings.

**Table 1:** The variation of FL crossover, super-Arrhenius crossover and effective activation energies from Arrhenius temperature dependence of D as a function of different spacings.

H	FL crossover	E_*a*_/k_*B*_	super-Arrhenius crossover
Bulk	≈600 K	680.3	—
70 Å (≈21*σ*)	≈600 K	735.7	—
30 Å (≈9*σ*)	≈800 K	996.4	—
20 Å (≈6*σ*)	≈800 K	1047.5	260 K
10 Å (≈3*σ*)	≈1000 K	1258.9	320 K
6.8 Å (=2*σ*)	≈1100 K	1502.6	360 K

For comparison, bulk values are also presented.

The FL crossover temperatures, obtained from the trends shown in Fig. 8, are found to be consistent with the other methods of identification, namely from DoS and from layer height analysis. The activation energy barriers for diffusion parallel to the walls are found to increase as we go from bulk to the narrower confined spacings. The Arrhenius to super-Arrhenius crossover temperature is found to be shifted to higher values as the spacings become smaller (Table 1).

The super-Arrhenius diffusion is characterized by a convex-shaped variation of D as a function of temperature^25,28^. From our earlier work^8^, we recall that glass-like behavior was emerging over intermediate timescales in a confined SCF with narrow spacing at room temperature (300 K), giving rise to a small D over long timescales. In the present work on confined SCF as a function of temperature, super-Arrhenius self-diffusion coefficients have been found over long timescales. The metastable configurations arising at these temperatures appear to have unusually large excess entropies as well. These features have been noted by M. Dzugutov^29^ who investigated the negative deviation from Arrhenius behavior of self-diffusion of metastable hard sphere liquids and associated this anomalous slowing down with configurational barriers governed by the excess entropy of the fluid.

To elucidate the super-Arrhenius diffusion at low temperatures at small spacings, we seek to investigate the correlation between structure and dynamics of SCF parallel to the walls. We compute the lateral-radial distribution function (${g}_{\parallel }$(r)) of the layer closest to the walls along with the two-body excess entropies s^(2)^, for different confined spacings, from the following equations^30^

7 $${g}_{\parallel }(r)\equiv \frac{1}{{\rho }^{2}V}\,\sum _{i\ne j}\,\delta (r-{r}_{ij})[\theta (|{z}_{i}-{z}_{j}|+\frac{\delta z}{2})-\theta (|{z}_{i}-{z}_{j}|-\frac{\delta z}{2})]$$

and

8 $${s}^{(2)}=-\,2\pi \rho {k}_{B}\,{\int }^{}[{g}_{\parallel }(r)\mathrm{ln}\,[{g}_{\parallel }(r)]-{g}_{\parallel }(r)+1]{r}^{2}dr$$

where V is the volume, $\rho$ is the number density, r_*ij*_ is the distance parallel to the walls between molecules i and j, z_*i*_ is the z coordinate of the molecule i, and $\delta (x)$ is the Dirac *δ* function. The Heaviside function $\theta (x)$ restricts the sum to a pair of particles located in the same slab of thickness $\delta z$, which have been considered to be same as the width of the layer closest to the wall.

**Figure 9:**
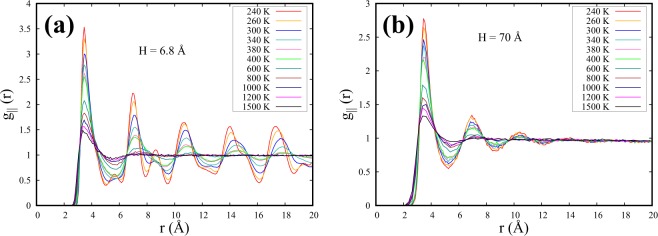
${g}_{\parallel }$(r) for the layer closest to the wall for (**a**) H = 6.8 Å and for (**b**) H = 70 Å as a function of temperature.

Figure 9 presents the ${g}_{\parallel }$(r) for the layer closest to the wall as a function of temperature for two spacings: H = 6.8 Å and H = 70 Å. For H = 6.8 Å (Fig. 9(a)), amorphous structural features with multiple distorted peaks in ${g}_{\parallel }$(r) are seen at lower temperatures. As the temperature is increased, the amorphous structural features gradually transform into liquidlike structures with the gradual decrements of the peak heights. After crossing FL, SCF goes into gaslike regime and is being reflected in the ${g}_{\parallel }$(r) with almost vanishing second and third peak heights. In a quite contrasting way, ${g}_{\parallel }$(r) for H = 70 Å shows a clear transformation exhibiting liquidlike structural features at low temperatures and gaslike structural features at high temperatures after crossing FL in between.

**Figure 10:**
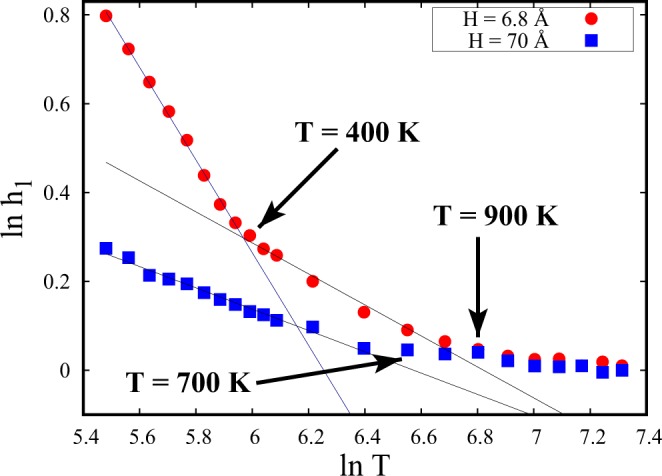
Variation of the heights (h_1_) of second coordination peaks of ${g}_{\parallel }$(r) (Fig. 9) as a function of temperature for H = 6.8 Å and H = 70 Å. The straight lines represent linear fits in the liquidlike regime of SCF. The data points are represented in log-scale.

In a recent study, L. Wang *et al*.^12^ proposed a power-law behavior of the temperature variation of the peak heights of g(r) in the liquidlike regime of bulk SCF and estimated the FL crossover based on the structural crossover of SCF. Taking cue from this work, the peak heights (h_1_) of the second coordination peak of ${g}_{\parallel }$(r) as a function of temperature are presented as a log (h_1_) − log (T) plot in Fig. 10 for the two spacings described above.

For H = 70 Å, the peak heights for the whole range of liquidlike regime are seen to be well fitted by a single straight line with a negative slope. The deviation at higher temperatures corresponding to the FL crossover is noted at ≈700 K for H = 70 Å and at ≈900 K for H = 6.8 Å, making it consistent with Table 1.

What seems interesting at H = 6.8 Å, is that the data for entire lower temperature range, before crossing FL, does not follow the same power law behavior. It is observed that two straight lines with different slopes are required to fit the whole range of peak heights before crossing FL. The two straight lines cross each other at a temperature where the structural features parallel to the walls start showing amorphous nature for H = 6.8 Å. Further, we observe that this feature bears a direct correlation to the super-Arrhenius diffusion discussed above. The crossing is happening at ≈400 K (Fig. 10) which is approximately the super-Arrhenius crossover temperature of diffusion coefficient at H = 6.8 Å, as tabulated in Table 1. This hints at the strong structure-dynamics correlation behind the emergence of super-Arrhenius dynamics of SCF parallel to the walls.

**Figure 11:**
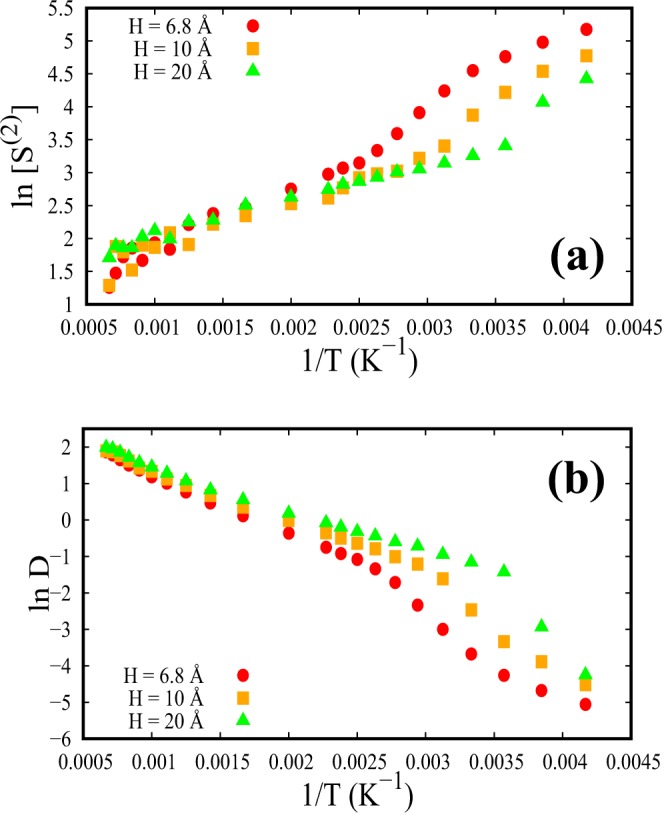
Correlation between D and scaled two-body excess entropy S^(2)^ (=$-{s}^{(2)}/2\pi \rho {k}_{B}$) of SCFs parallel to the walls are represented as a function of inverse of temperature for different confined spacings. (**a**) ln [S^(2)^] as a function of 1/T. (**b**) ln D as a function of 1/T.

To further understand the structure-dynamics correlation we look at the pair-excess entropy of SCF parallel to the walls. For a fixed temperature, the density for bulk as well as confined systems with variable spacings is kept fixed. So, we scale s^(2)^ and use $\frac{{s}^{(2)}}{2\pi \rho {k}_{B}}$ for all the calculations involving the two-body excess entropy. The values of $\frac{-{s}^{(2)}}{2\pi \rho {k}_{B}}$ are found to be higher for confined SCFs compared to the bulk system, throughout the whole range of temperature, as a result of increased ordering under confinement. More interestingly, Fig. 11(a) marks a significantly unusual behavior at low temperatures with an unprecedented enhancement of excess entropy parallel to the walls at smaller spacings. This abrupt enhancement of structural ordering associated with the pair-excess entropy with decreasing H seems to have a strong correlation with super-Arrhenius diffusion parallel to the walls. The super-Arrhenius diffusion at low temperatures, before crossing the FL, shown in Fig. 11(b), is found to be a direct consequence of an enhanced ordering, evidenced by the enhancement of $-{s}^{(2)}/2\pi \rho {k}_{B}$. This sudden jump in entropy of SCF parallel to the walls, of the layer closest to the wall, supports the argument^29^ of configurational excess entropy barrier causing a slowdown in the dynamics at low temperatures with small spacings.

We note here that both the Arrhenius and the super-Arrhenius behavior of self-diffusivity of SCF parallel to the walls are long timescale features. Interestingly, this long time slowing down (super-Arrhenius) characteristics at low temperatures for smaller spacings stem from the dynamics and structural features of SCF parallel to the walls at intermediate timescales.

**Figure 12:**
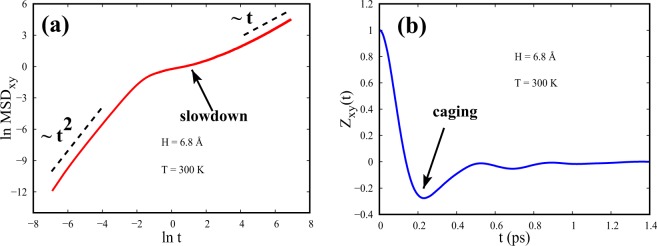
(**a**) ${{\rm{MSD}}}_{\parallel }$ (MSD along x, y) and (**b**) Normalized ${{\rm{VACF}}}_{\parallel }$ (denoted as Z_*xy*_) as a function of time, at T = 300 K and P = 5000 bar of confined supercritical Argon with spacing = 6.8 Å.

Velocity autocorrelation function (VACF) and mean squared displacement (MSD) in Fig. 12 show such an example of a glass-like slowdown at the intermediate timescales for small spacing and at a low temperature. Specifically, at T = 300 K and for a spacing of 6.8 Å (=2*σ*), corresponding to a temperature and spacing where super-Arrhenius diffusion is seen to exist, a prominent slowdown and a strong caging effect can be seen over intermediate timescales as shown from ${\mathrm{MSD}}_{\parallel }$ and ${\mathrm{VACF}}_{\parallel }$ respectively in Fig. 12(a,b). After a certain temperature, the continuous change of energy barriers owing to the continuous variation of configurational excess entropy with temperature at smaller spacings eventually lead to the long time slowing down of self-diffusivity featuring super-Arrhenius trend.

## Summary

In summary, we study the temperature evolution of the structure and dynamics of confined supercritical LJ fluids at a constant pressure and compare the FL crossover characteristics to that of the bulk SCF using a series of molecular dynamics simulations. Five different spacings have been considered in this study. For each spacing, confined SCF is simulated using NVT ensemble, for temperatures ranging from 240 K to 1500 K keeping the pressure fixed at 5000 bar. The densities of the confined fluids are maintained to be identical to that in the bulk phase corresponding to the chosen P and T. Atomistic walls are modeled perpendicular to the z-axis and periodic boundary conditions are employed along the other two directions (x, y).

Using DoS function parallel to the walls (Z_*xy*_($\omega$)), we characterize the crossover from liquidlike damped oscillatory microscopic dynamics to gaslike non oscillatory diffusion dominated dynamics for confined fluids with variable spacings. The partially confined SCFs are seen to exhibit a progressive shift of FL crossover point to higher temperatures for smaller spacings compared to the bulk SCF. The vanishing of the peak in Z_*xy*_($\omega$) indicates that the fluid no longer supports shear mode excitations and serves as an identifier of the FL crossover in confined SCF just as is done for the bulk in literature. Further, monitoring the intercept of Z_*xy*_($\omega$) at $\omega =0$, reveals a gradual increase in diffusive modes as temperature is increased. As we decrease the spacing between the walls, wall-mediated collisions seem to influence Z_*xy*_($\omega$) strongly. Almost negligible diffusive modes and weak shoulder in the Z_*xy*_($\omega$) at higher frequencies carry this signature.

The layering of fluid particles across the spacing also feature this crossover characteristics. Analyzing the systematic decay of the peak height of the layer closest to the wall as a function of temperature, consistent FL crossover temperatures are obtained for different spacings. This shift in the FL, specially the persisting oscillatory nature of the dynamics up to a reasonably higher temperature at small spacings, suggests an intriguing possibility of an unusual temperature dependence of diffusion processes in confined SCF across FL.

Indeed, we observe two distinct crossover phenomena in the evolution of D as a function of temperature along the isobaric line of P = 5000 bar. Firstly, in the liquidlike regime of SCF, diffusion coefficient obeys Arrhenius law until a high temperature which we consider as the FL crossover point. After crossing FL the fluid dynamics becomes purely diffusive unlike the liquidlike regime where both caging and diffusion prevail. The activation energies of free energy barriers are calculated from the slope of the Arrhenius fit of diffusivity in the liquidlike regime.

The FL crossover has also been monitored from the deviation of Arrhenius behavior of D at high temperatures. Further, at low temperatures, self-diffusion coefficients of confined supercritical fluids are found to markedly deviate from Arrhenius behavior and exhibit super-Arrhenius behavior for smaller spacings. This crossover from Arrhenius to super-Arrhenius diffusivity shifts towards higher temperatures with decreasing spacings. We also study the structural features parallel to the walls using the temperature evolution of ${g}_{\parallel }$ (r) peaks, specifically the second coordination peaks to detect FL crossover temperatures. We found the FL crossover of confined SCF using ${g}_{\parallel }$ (r) peaks to be consistent with the other methods followed earlier. Further, super-Arrhenius crossover has also been detected using the temperature evolution of the ${g}_{\parallel }$ (r) peaks.

Moreover, a strong correlation between diffusion and excess entropy has been found for smaller spacings parallel to the walls. This enhanced ordering, captured by the excess entropy at low temperatures, affects the long timescale dynamics of the fluid by reducing the diffusion coefficient. Thus excess entropy has been found to play a crucial role in slowing down the fluid dynamics. This long timescale slowdown in SCFs is similar to glass-forming liquids or binary supercooled fluids where super-Arrhenius diffusion had been found to exist^25,29^.

The origin of glass-like long timescale diffusion of confined SCFs at low temperatures, has emerged out of the interplay between structure and dynamics on intermediate timescales. The MSD and VACF feature characteristic slowdown and strong caging phenomena over intermediate timescales.

The changes in the FL for the confined SCFs compared to that of the bulk phase are found to show rich dynamics and structural features. The interplay of temperature and confinement in modifying the dynamics and structure of SCF may enable tuning of their properties to suit specific applications. It would be interesting to examine diffusion of’doped’ ions/atoms in such tunable systems.

## Supplementary information

Supplementary material
