# Supplementary material for: Frenkel line crossover of confined supercritical fluids
Source: Sci Rep. 2019 Oct 16;9:14872. doi: 10.1038/s41598-019-49574-3 (PMC6795815; doi:10.1038/s41598-019-49574-3)
Supplement: Supplementary file 1 — Supplementary material [file 41598_2019_49574_MOESM1_ESM.pdf]

Supplementary material: Frenkel line crossover of confined supercritical fluids

Kanka Ghosh* and C.V.Krishnamurthy†

Department of Physics, Indian Institute of Technology Madras, Chennai-600036, India

I. PEAK HEIGHT VARIATION OF LAYER CLOSEST TO THE WALL

Figure S1 depicts the temperature evolution of the peak heights (h) for the layer closest to the wall for two extreme spacings H = 6.8 Å and H = 70 Å. The peak height of the layer closest to the wall is observed to decay faster for H = 6.8 Å compared to the H = 70 Å spacing.

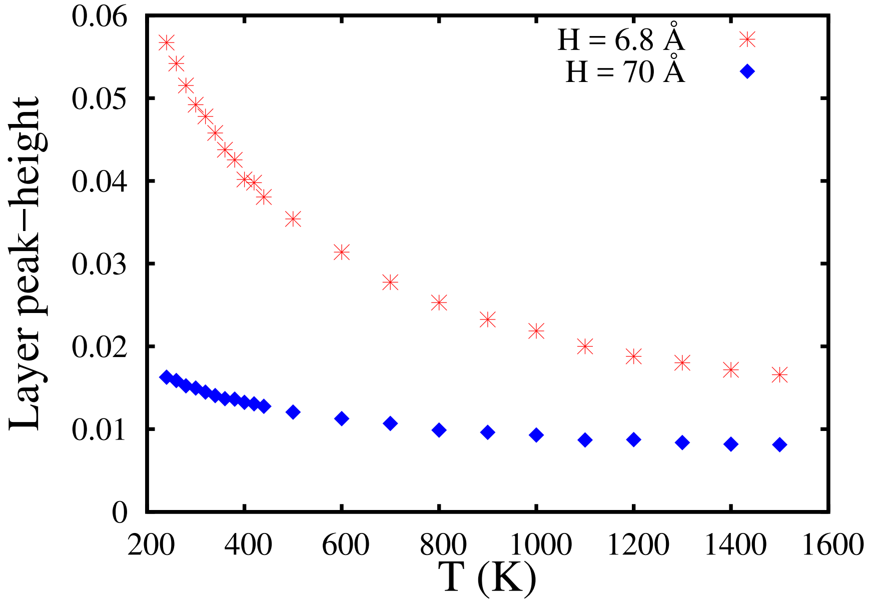

FIG. S1. Peak height of the layer close to the wall as a function of temperature for H = 6.8 Å and 70 Å spacings.

II. VARIATION OF SELF-DIFFUSION COEFFICIENTS (D), PARALLEL TO THE WALLS, AS A FUNCTION OF INVERSE OF TEMPERATURE FOR DIFFERENT CONFINED SPACINGS.

Figure S2 shows the variation of self-diffusion coefficients (D) of bulk supercritical LJ fluid, parallel to the walls, as a function of inverse of temperature for different confined spacings (H). The trends of D for different confined spacings (H) are seen to diverge for lower temperatures (Fig S2).

* kankaghosh@physics.iitm.ac.in

† cvkm@iitm.ac.in

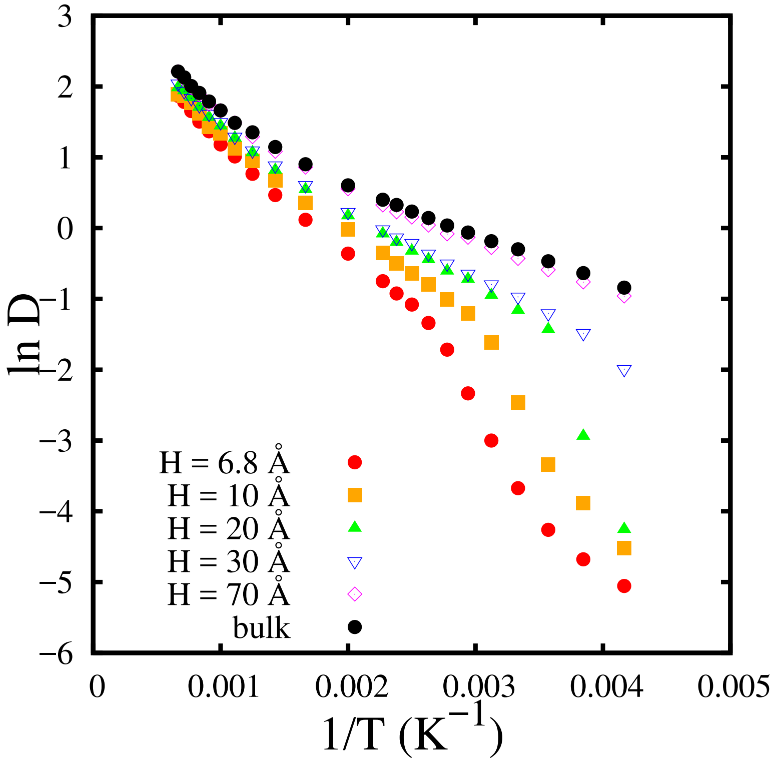

FIG. S2. Self-diffusion coefficients (D) of SCFs, parallel to the walls, as a function of inverse temperature, for different H. For comparison bulk self-diffusion coefficient is also included. A logarithmic scale is chosen for representing D to identify Arrhenius behaviour.

III. DENSITY OF STATES PARALLEL ($Z_{xy}(\omega)$) AND PERPENDICULAR ($Z_z(\omega)$) TO THE WALLS AS A FUNCTION OF TEMPERATURE FOR H = 6.8 Å SPACING

Figure S3 shows $Z_{xy}(\omega)$ and $Z_z(\omega)$ as function of temperature for H = 6.8 Å (= 2σ) spacing. For all temperatures shown in the figure (240 K $\leqslant$ T $\leqslant$ 500 K) $Z_z(\omega)$ shows strong oscillatory modes due to the effect of confinement. We note that throughout the temperature range shown in Fig S3, $Z_z(\omega) = 0$ at $\omega = 0$, which reflects the non-diffusive motion normal to the walls. It is also observed that at very low temperatures, $Z_z(\omega)$ exhibits two closely separated peaks. A gradual change in $Z_z(\omega)$ from the two-peak structure to single peak structure is seen as we increase the temperature. It is interesting to note that at very low temperatures, in such strong confinement (H = 2σ), $Z_z(\omega)$ significantly influence the $Z_{xy}(\omega)$. We find that $Z_{xy}(\omega)$ develops a shoulder at a higher frequency which coincides with the frequency of the second peak in $Z_z(\omega)$.

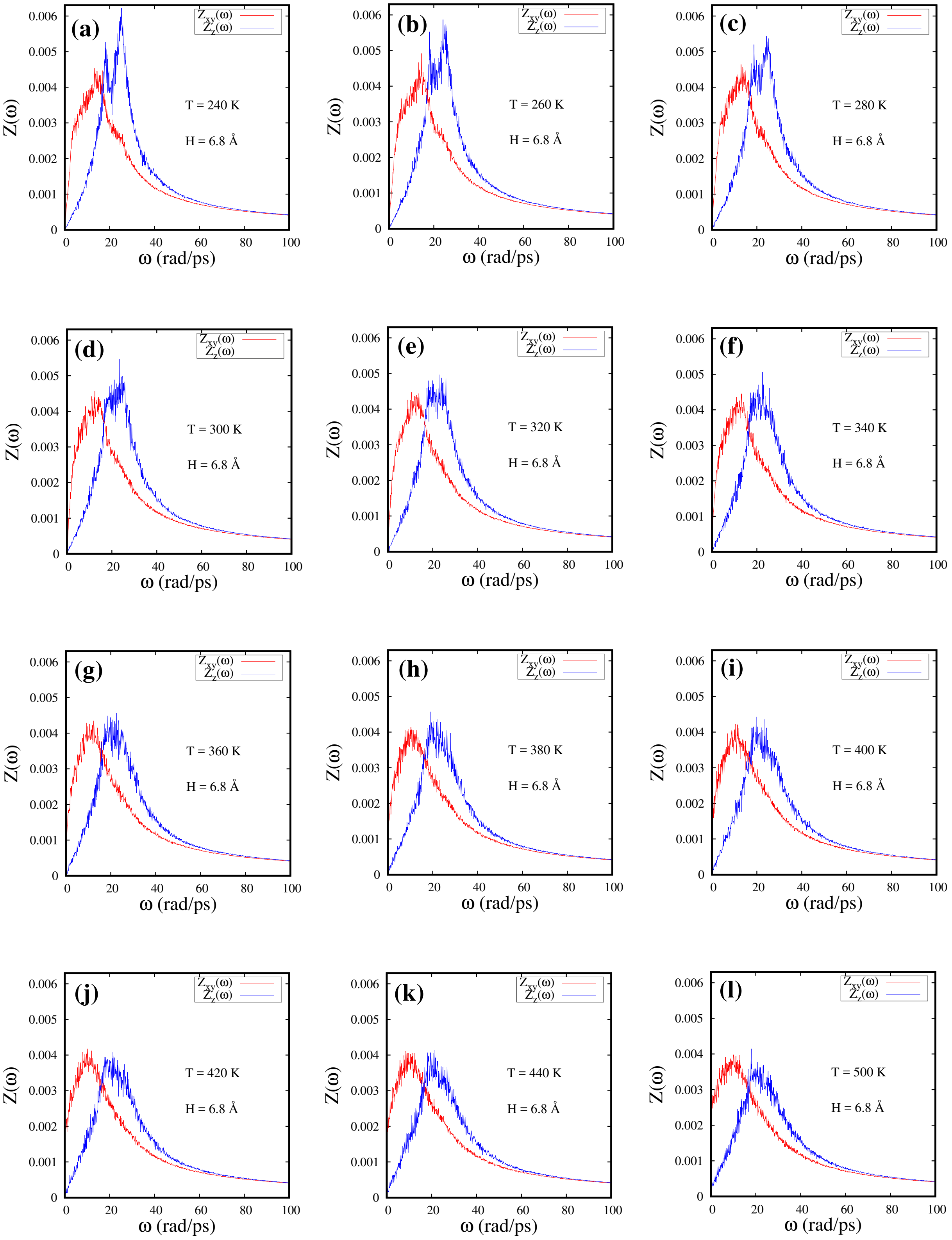

FIG. S3. DoS parallel ($Z_{xy}(\omega)$) and perpendicular to the walls ($Z_z(\omega)$) of SCF is presented for different temperatures for H = 6.8 Å system. The corresponding temperatures are (a) 240 K, (b) 260 K, (c) 280 K, (d) 300 K, (e) 320 K, (f) 340 K, (g) 360 K, (h) 380 K, (i) 400 K, (j) 420 K, (k) 440 K and (l) 500 K.

This shoulder in $Z_{xy}(\omega)$ seems to exist for T = 240 K, 260 K and 280 K, where two-peak structures of $Z_z(\omega)$ are also found to be present. Therefore, we find that at low temperatures and in a strongly confined SCF, wall-mediated collisions influence the the parallel component of DoS significantly.
